# Supplementary material for: Antioxidant Effects and Phytochemical Properties of Seven Taiwanese Cirsium Species Extracts
Source: Molecules. 2021 Jun 28;26(13):3935. doi: 10.3390/molecules26133935 (PMC8272034; doi:10.3390/molecules26133935)
Supplement: Supplementary file 1 [file molecules-26-03935-s001.zip › molecules-1240682-supplementary.pdf]

Antioxidant Effects and Phytochemical Properties of Seven Taiwanese *Cirsium* Species Extracts

Zi-Wei Zhao [1,†], Hung-Chi Chang [2,†], Hui Ching [3], Jin-Cherng Lien [4], Hui-Chi Huang [5] and Chi-Rei Wu [5,*]

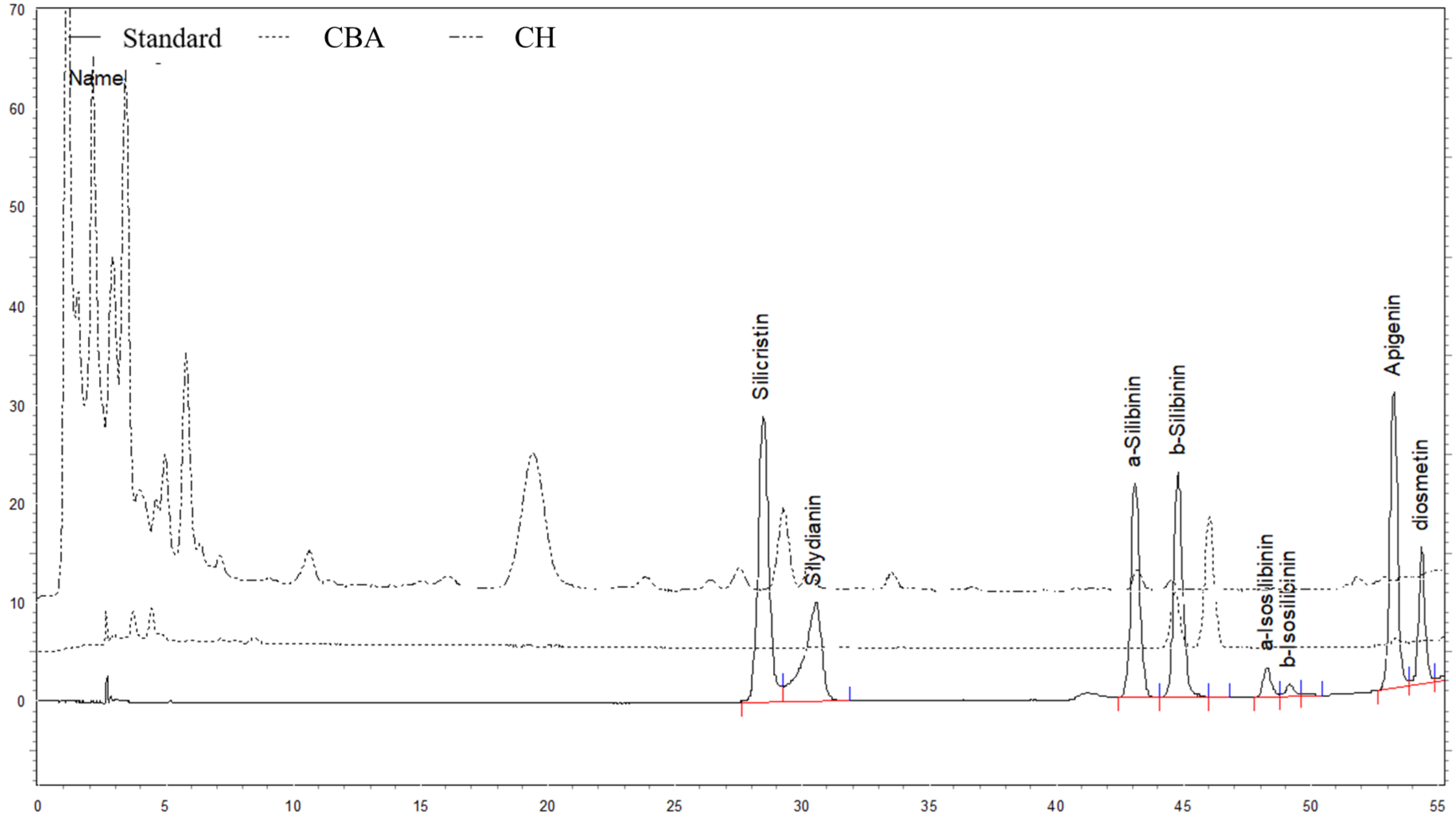

Figure S1. High-performance liquid chromatography (HPLC) chromatogram of standards, the methanolic standard extracts of CBA and CH at 280 nm. CBA, the aerial part of *Cirsium brevicaule*; CH, Cirsii Herb.

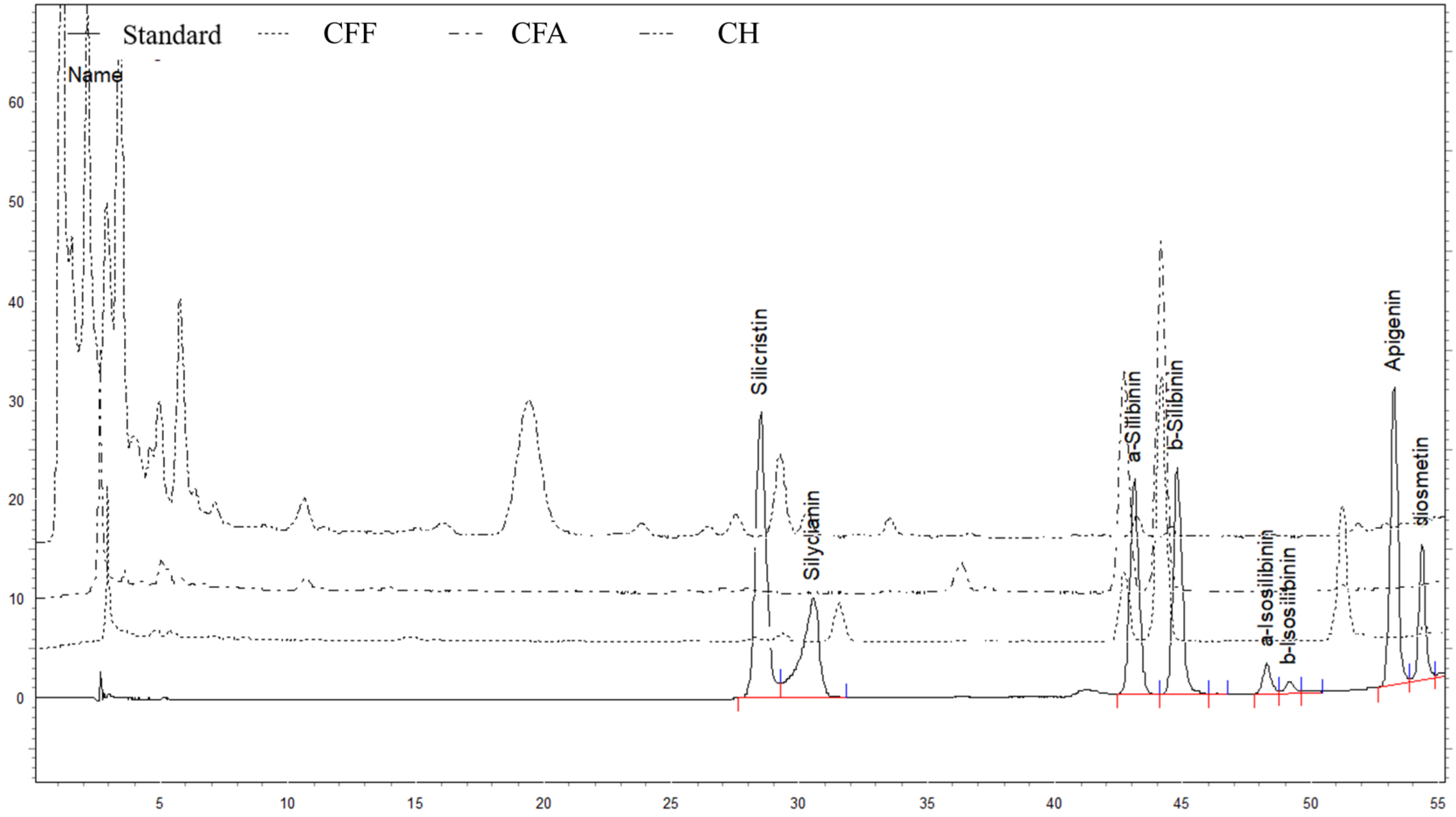

Figure S2. High-performance liquid chromatography (HPLC) chromatogram of standards, the methanolic standard extracts of CFA, CFF and CH at 280 nm. CFA, the aerial part of *Cirsium ferum*; CFF, the flower part of *Cirsium ferum*; CH, Cirsii Herb.

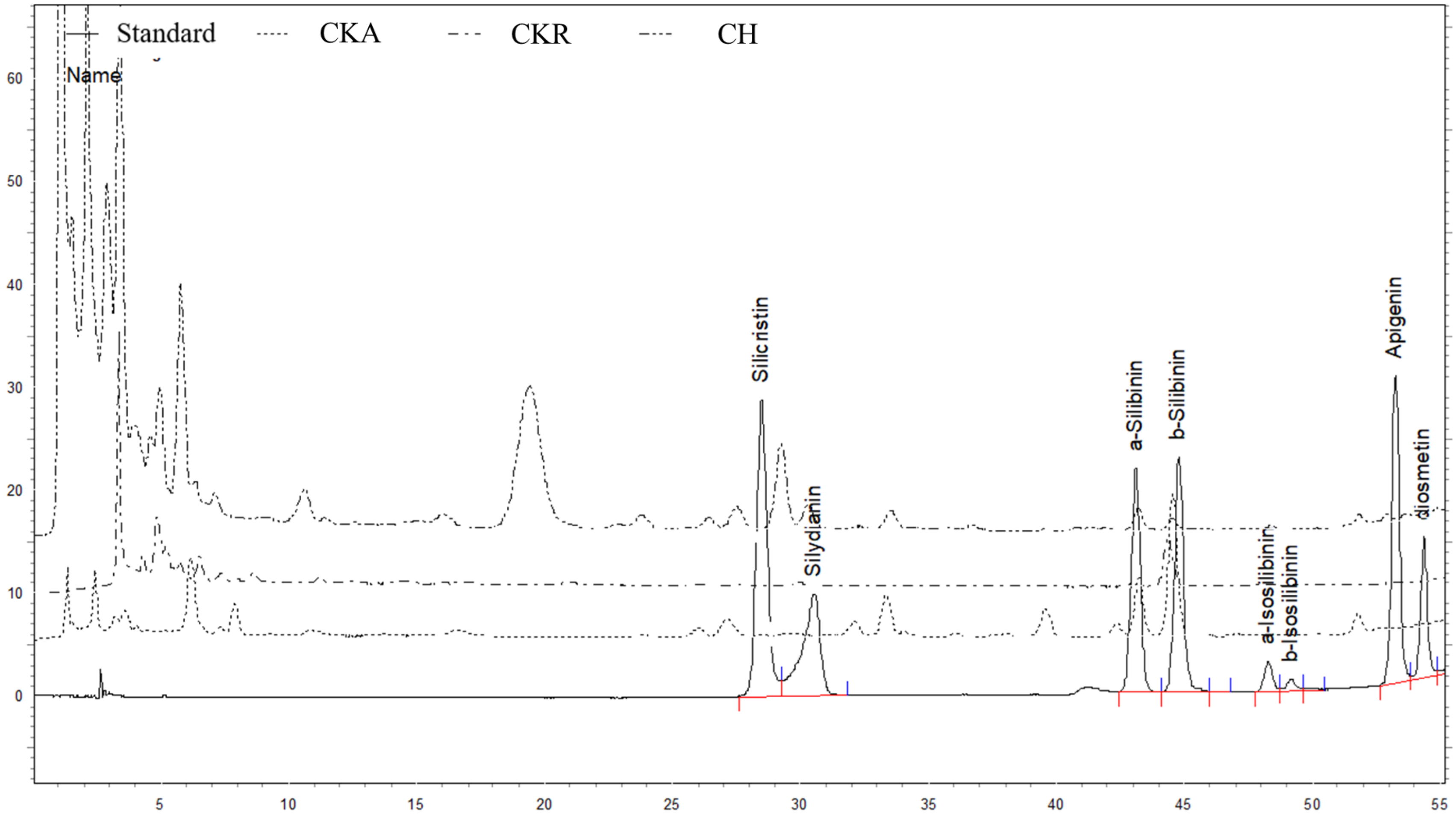

Figure S3. High-performance liquid chromatography (HPLC) chromatogram of standards, the methanolic standard extracts of CKA, CKR and CH at 280 nm. CKA, the aerial part of *Cirsium kawakamii*; CKR, the radix part of *Cirsium kawakamii*; CH, Cirsii Herb.

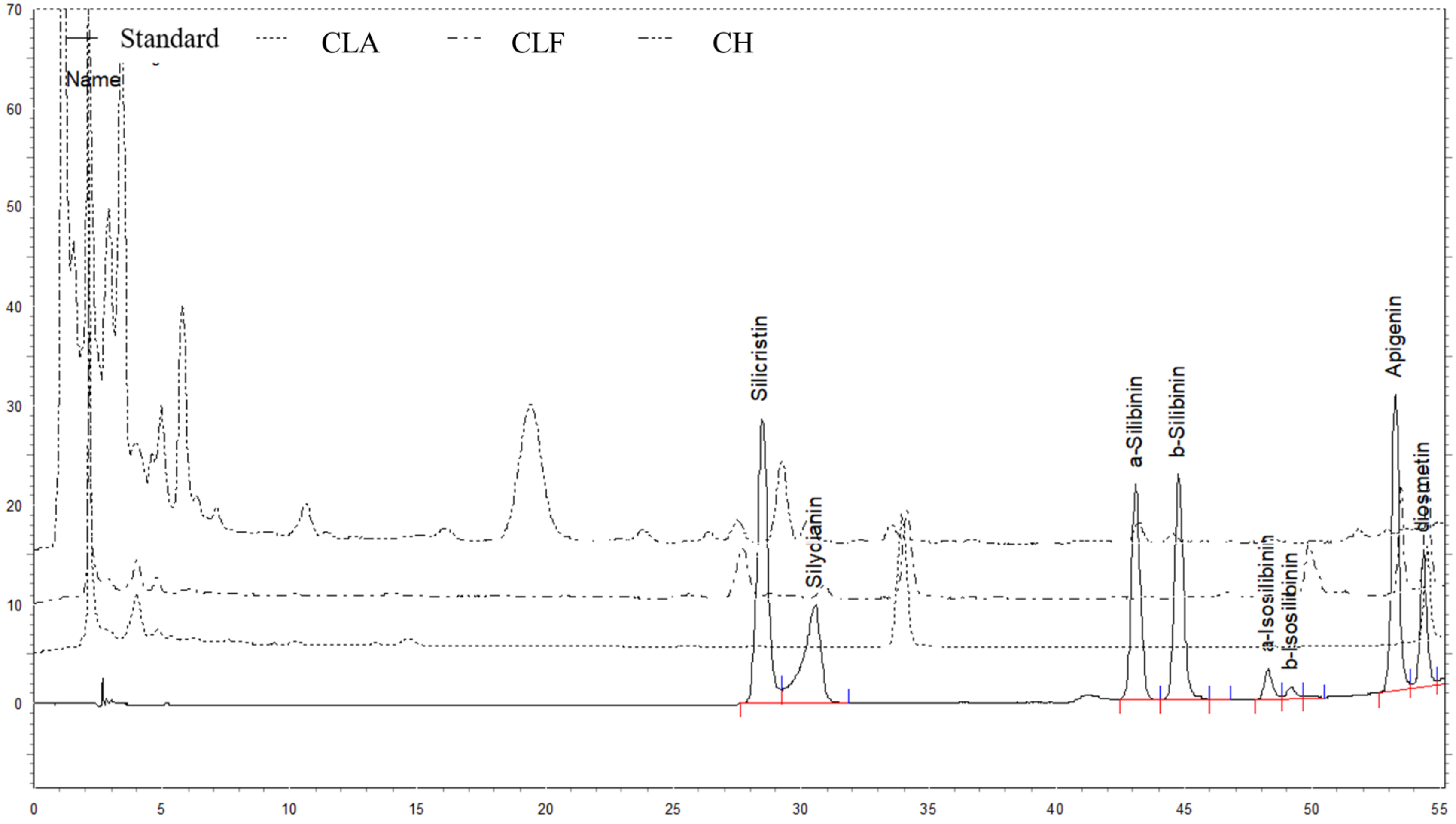

Figure S4. High-performance liquid chromatography (HPLC) chromatogram of standards, the methanolic standard extracts of CLA, CLF and CH at 280 nm. CLA, the aerial part of *Cirsium lineare*; CLF, the flower part of *Cirsium lineare*; CH, Cirsii Herb.
